# Local–Global Feature Adaptive Fusion Network for Building Crack Detection

**DOI:** 10.3390/s24217076

**Published:** 2024-11-03

**Authors:** Yibin He, Zhengrong Yuan, Xinhong Xia, Bo Yang, Huiting Wu, Wei Fu, Wenxuan Yao

**Affiliations:** 1 Hunan Architectural Design Institute Group Co., Ltd., Changsha 410082, China; ybhe@hnu.edu.cn (Y.H.); xiaxh@hnadi.com.cn (X.X.); yangbo@hnadi.com.cn (B.Y.); 2 College of Electrical and Information Engineering, Hunan University, Changsha 410082, China; wu_948@hnu.edu.cn (H.W.); wenxuanyao@hnu.edu.cn (W.Y.); 3 College of Information Science and Engineering, Hunan University, Changsha 410082, China; fuwei@hnu.edu.cn

**Keywords:** crack detection, CNN, Mamba, multi-feature adaptive fusion

## Abstract

Cracks represent one of the most common types of damage in building structures and it is crucial to detect cracks in a timely manner to maintain the safety of the buildings. In general, tiny cracks require focusing on local detail information while complex long cracks and cracks similar to the background require more global features for detection. Therefore, it is necessary for crack detection to effectively integrate local and global information. Focusing on this, a local–global feature adaptive fusion network (LGFAF-Net) is proposed. Specifically, we introduce the VMamba encoder as the global feature extraction branch to capture global long-range dependencies. To enhance the ability of the network to acquire detailed information, the residual network is added as another local feature extraction branch, forming a dual-encoding network to enhance the performance of crack detection. In addition, a multi-feature adaptive fusion (MFAF) module is proposed to integrate local and global features from different branches and facilitate representative feature learning. Furthermore, we propose a building exterior wall crack dataset (BEWC) captured by unmanned aerial vehicles (UAVs) to evaluate the performance of the proposed method used to identify wall cracks. Other widely used public crack datasets are also utilized to verify the generalization of the method. Extensive experiments performed on three crack datasets demonstrate the effectiveness and superiority of the proposed method.

## 1. Introduction

With the rapid development of the economy and the acceleration of urbanization, buildings have been greatly and widely built. However, building structures are subject to varying degrees of deterioration over long periods, which are prone to cracks after being eroded by the external environment, such as high temperatures, ultraviolet rays, and rain, causing the bearing capacity of the buildings to decline. The cracks will seriously affect the safety and stability of each component of the buildings [1]. If the cracks are not repaired promptly, they may continue to expand and spread to the internal structure of the buildings, drastically reducing the life of the buildings. Once the building collapses due to damage, it will greatly threaten the safety of people’s property and lives, eventually causing irreversible damage [2]. Therefore, buildings must be regularly inspected for health so that these cracks can be detected in time and handled with reasonable means, which ensures the safety and stability of the building structures [3].

The traditional manual inspection methods are inefficient and have safety hazards, which are not applicable to various high-rise buildings, while the use of drone technology will solve this problem to a large extent due to its high mobility. Combining the UAV photography technology with the automated crack detection technology is expected to achieve high efficiency, high precision, and high safety factors in obtaining and analyzing crack information [4].

In general, digital image processing-based methods, machine learning-based methods, and deep learning-based methods are the three main types of methods for crack detection. The methods based on digital image processing techniques can be categorized into threshold segmentation methods [5,6] and edge detection algorithms [7,8]. The core of the threshold segmentation method lies in choosing a threshold value and comparing the threshold value with the pixel value to determine which category the pixel belongs to, which is commonly used to segment images into different regions or objects. For instance, Oliveira et al. [9] utilized the dynamic threshold method to identify concrete cracks from small-sized image blocks for automatic crack classification. Peng et al. [10] proposed a crack detection method based on a quadratic threshold segmentation technique to detect cracks on airport runways. The edge detection algorithm uses a differential function to identify the edge information based on the large difference in gray values between the crack edge and the background. The Sobel algorithm was introduced by Dhule et al. [11] to identify building cracks in UAV images to assist engineers in maintaining buildings. Although the methods based on digital image processing are simple, they are very much affected by the environment, lack robustness when faced with complex conditions, and are not highly automated. In contrast, machine learning and deep learning can automatically learn the information in the image, which improves the damage detection accuracy as well as the robustness in complex scenes, and gradually become the mainstream damage detection technology.

Machine learning (ML), as a core branch of artificial intelligence (AI), has shown great potential and application value in several fields in recent years. Machine learning enables computer systems to learn from large amounts of historical data to generate empirical models, and then guide business decisions and make predictions. Machine learning contains a variety of algorithms, including decision trees [12], plain Bayesian classifiers [13], random forests [14], support vector machines [15], and neural networks [16]. For example, Moon et al. [17] proposed a back-propagation neural network for the identification of surface cracks in concrete structures. Unfortunately, traditional machine learning methods rely on specialized knowledge and experience, restricting its widespread application. Therefore, the researchers focus on deep learning which can learn more complex patterns and laws from large amounts of data.

Deep learning can obtain the patterns in pictures from a large amount of data automatically and extract more abstract features by utilizing multi-layer neural networks for training. Deep learning-based methods significantly improve detection accuracy, enabling them to adapt to more complex scenarios and improve both generalization and robustness. As a result, they have a widespread application in crack detection. In particular, the convolutional neural network (CNN) methods [18,19] are widely used due to their ability to efficiently capture local features. However, these methods cannot acquire the global dependencies of the images, making it difficult to identify complete cracks, which manifests in that the fracture phenomenon of cracks is easily caused. Therefore, more and more transformer-based methods [20,21] have been proposed, which have the ability to model the long-range dependencies by utilizing the self-attention mechanism. Unfortunately, high computational complexity and low efficiency limit their development.

In order to address the aforementioned limitations, a local–global feature adaptive fusion network, referred to as LGFAF-Net, is proposed in this article. The model solves the limitations of the transformer in terms of computational complexity to a certain extent while retaining its ability to capture global features. Meanwhile, the convolutional neural network structure preserves the capability to extract local information. Therefore, the cracks are identified more accurately and completely. Specifically, the main contributions of this paper are as follows:

1. A local–global feature adaptive fusion network, referred to as LGFAF-Net, is proposed by introducing the VMamba encoder and the residual network. The CNN specializes in local feature extraction and the VMamba can efficiently capture global long-range dependencies and spatial structure in an image, where combining the two allows the model to better understand the overall structure and contextual information of the image while preserving the image details.
2. For better integration of the global and local features from the dual-encoding, a multi-feature adaptive fusion module is designed to facilitate the learning of representative features and enhance crack detection capabilities effectively by combining the self-attention mechanism [22] and the spatial and channel reconstruction convolution (SCConv) [23].
3. To compensate for the lack of the building exterior wall cracks, the BEWC dataset captured by UAVs is proposed to measure the performance of the proposed method used to identify wall cracks.

## 2. Related Work

Currently, CNNs in deep learning methods are capable of effectively extracting deep-level features so that they can be widely applied in segmentation and crack detection. Long et al. [24] proposed the fully convolutional network (FCN) built with an end-to-end CNN architecture, marking the pioneering work of deep learning in semantic segmentation. Ronneberger et al. [25] designed the U-Net with a symmetric encoder–decoder structure and skip connections to reduce information loss and preserve spatial information at different scales. Chen et al. [26] designed atrous spatial pyramid pooling (ASPP) to enlarge the receptive field and proposed the Deeplab series of network structures, significantly enhancing segmentation accuracy. SegNet, [27], which is grounded in the principles of fully convolutional networks (FCNs), incorporates a symmetric encoder–decoder structure with a pooling index transmission mechanism. Based on SegNet, Zou et al. [28] introduced multi-scale fusion to construct the Deepcrack network for crack segmentation, further enhancing the precision of crack extraction. Xu et al. [29] proposed a modified fusion convolutional neural network aimed at boosting the accuracy of crack detection. Ren et al. [30] employed CNNs to build an encoder–decoder network structure, incorporating atrous convolution and spatial pyramid pooling to increase the receptive field to some extent and capture multi-scale information from images. This enables the network to acquire more extensive contextual information, further enhancing its performance. Based on pre-trained ResNet34, Lin et at. [31] proposed the EMRA-Net with a pyramid edge module and a multi-scale fusion module. Through self-learning weighting in the multi-scale fusion module, the network effectively enhances the identification of subtle defects. Ma et al. [32] introduced separable convolutions into crack detection to reduce model parameters, achieved multi-scale feature enhancement through cross-layer feature learning and fusion, and utilized a hybrid multi-attention module to capture long-term relationships in cracks to enhance crack recognition capabilities. Although these CNN-based methods have achieved satisfactory results in crack detection, their relatively poor ability to capture global dependencies can easily lead to discontinuous and fragmented segmentation.

Vision transformer (ViT) [33] is an architecture that applies the transformer designed for natural language processing to the field of computer vision. It utilizes a global self-attention mechanism to process image data, aiming to overcome the limitations of CNN in capturing long-term dependencies within images. Swin transformer [34] adopts a hierarchical construction similar to CNN, building higher-level representations through multi-stage extraction and feature downsampling, which enables the model to better adapt to various visual tasks. Mobile ViT [35] is a lightweight vision transformer structure that reduces the number of model parameters while maintaining the ability to learn global representations. Segformer [36] eliminates the need for positional encoding, thereby avoiding performance degradation caused by them. In addition, it introduces a lightweight multilayer perceptron (MLP) structure and generates the final segmentation results by fusing information from different levels. TransUNet [37] combines the global context understanding capabilities of the transformer with the local feature extraction capabilities of U-Net. Through its hybrid architecture design, the model can balance both local details and global features, achieving better performance in image segmentation tasks. Liu et al. [38] designed a novel transformer encoder block, using it to construct an encoder–decoder network. They also proposed a scale-attention fusion module to emphasize semantic features. Bai et al. [39] leveraged both CNN and transformer to propose a dual-encoder multi-scale fusion network, which captures both global and local features. They further designed an interactive learning module to more effectively fuse features from different branches.

## 3. Methodology

In this section, a comprehensive overview of the proposed LGFAF-Net is presented. Firstly, the two-branch structure is introduced to capture local and global features, followed by a decoder unit that combines the visual state space block (VSS block) and the CNN, ultimately forming a U-shape structure network. In addition, the local and global features are fused by the multi-feature adaptive fusion. This section aims to explain each component’s functions and contributions.

### 3.1. Network Overview

In general, local information is important for capturing fine cracks, while long and complex cracks need to be identified completely with more global long-range dependencies, which demonstrates the necessary for crack segmentation to introduce a dual-encoding method based on the CNN and Mamba to extract local and global features.

**Figure 1:**
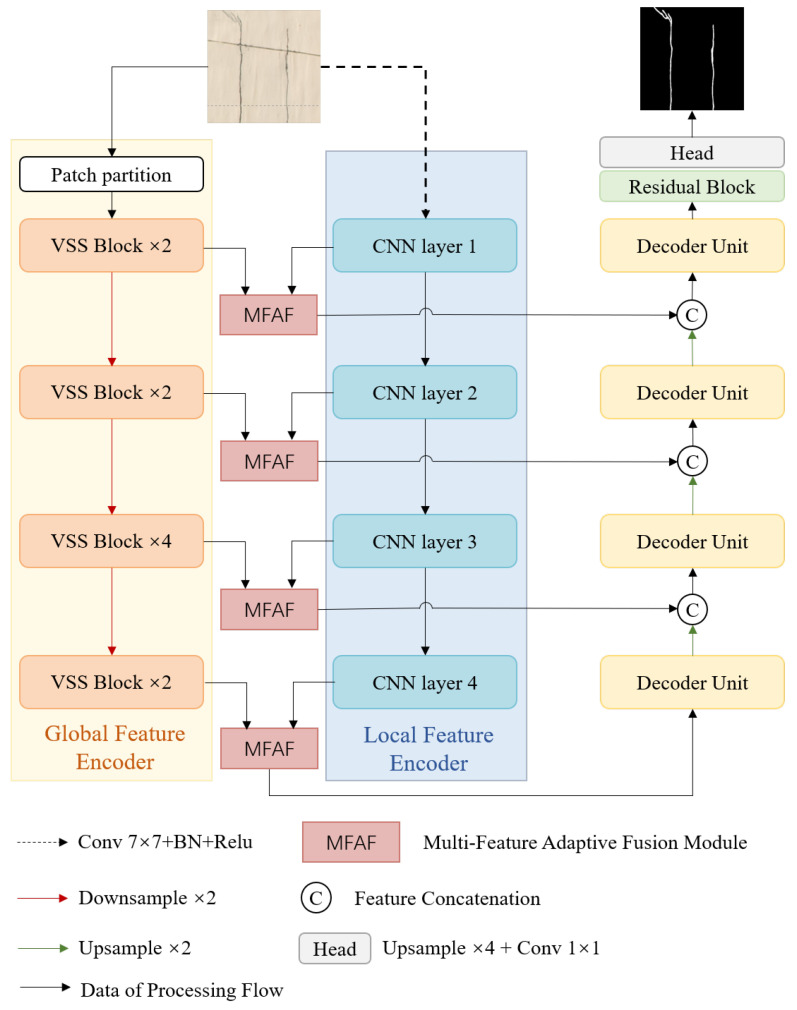
Network framework of proposed LGFAF-Net.

As shown in Figure 1, the proposed LGFAF-Net focuses on three main stages: (1) Encoding stage: To enhance the ability to capture the local information and global long-term dependencies, a dual-encoding branch based on the CNN and Mamba is built, where the local feature extraction branch is constructed with ResNet34 [40] to enhance local patterns and the global feature extraction branch is built with the VMamba encoder [41] to acquire global features. (2) Multi-feature adaptive fusion: To effectively fuse the local and global features from the two-branches, a multi-feature adaptive fusion module is produced with the self-attention mechanism and the SCConv. (3) Decoding stage: In this stage, the decoder unit is proposed with the visual state space block and the CNN for information reconstruction and feature mapping. Furthermore, a skip connection is introduced to preserve spatial information at different resolutions. Finally, a U-shape construction for crack identification is built.

### 3.2. Encoding Stage

For the feature extraction, the CNN network and Mamba model focus on different aspects, where the former is concerned with local patterns and the latter pays attention to global long-range dependencies. Through utilizing the CNN and Mamba as the dual-encoding branches, both local detailed information and global features are captured so as to increase the accuracy of crack recognition.

**Global Feature Encoder**: Mamba [42] is a new selectively structured state space model that excels in long sequence modeling tasks. To better suit 2D image tasks, researchers extend the concepts and architecture of the Mamba to computer vision, and the architecture of the VMamba is built through designing the innovative technology of the cross-scan module (CSM) for the adaptation of two-dimensional spatial features. In this paper, the VMamba encoder acts as the global feature encoder to enable the network to efficiently model the long-range dependencies of image data through its global sense field and dynamic weighting mechanism. Firstly, the VMamba divides the input image into blocks, preserving the two-dimensional structure of the image. There are four stages in the VMamba encoder, where each stage is constructed by stacking multiple VSS blocks, and the number of VSS blocks for the four stages is two, two, four, and two, respectively. Furthermore, the feature maps of the previous stage are subjected to a downsampling operation before being fed into the next stage, which ultimately results in the feature maps of the four stages with different resolutions.

**Figure 2:**
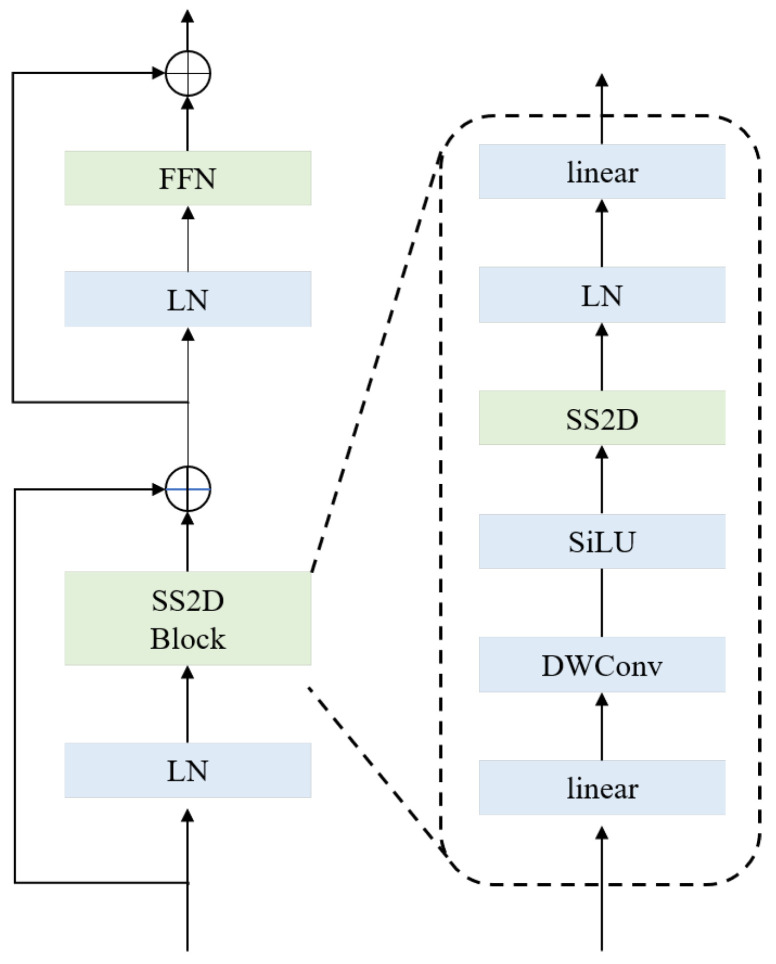
Network structure of the VSS block [41].

The core component of the VMamba is the visual state-space block, as shown in Figure 2. The VSS block consists of two residual modules to effectively mitigate the problems of gradient vanishing and gradient explosion through residual connection, making the network easier to optimize. To be specific, the input feature map is divided into two branches, one of which is fed into the 2D selective scan block (SS2D block) after the layer normalization, followed by an element-wise addition operation with the original features of the other branch. In the SS2D block, before being fed into the SS2D which is the most important component of the SS2D block, it is necessary for feature maps to pass through the linear layer, the DWconv, and the SiLU activation function to enhance the nonlinearity and improve the network’s performance. Another layer normalization function and linear layer are employed to map the input data into a new space to form a higher-level feature representation. After passing through the first residual block, the corresponding output features are fed into the second residual block. Similarly, the second residual block is also divided into two branches, one of which is fed into the feed-forward neural network (FFN) layer after the layer normalization, performing further feature extraction and transformation of the data, and then the element-wise addition operation is applied to fuse it and the features of the other branch to obtain the final output features of the VSS block.

**Local Feature Encoder**: The CNNs have made significant progress in crack detection due to their strong ability to capture detailed information, in which the residual network (ResNet) has been widely used. ResNet is designed to address the training challenges of deep convolutional neural networks when increasing the depth of the network, such as gradient vanishing or gradient explosion. ResNet greatly improves the training effect and depth of the network by introducing the concept of residual learning, allowing the network to learn the information between inputs and outputs.

In this article, ResNet34 is employed to build the CNN branch. Firstly, a 7 × 7 convolution layer is adopted to expand the receptive field. Then the features are fed into the next four layers built with residual units. The residual blocks contained in the residual units of the first to fourth layers are 3, 4, 6, and 3, respectively.

### 3.3. Multi-Feature Adaptive Fusion

**Figure 3:**
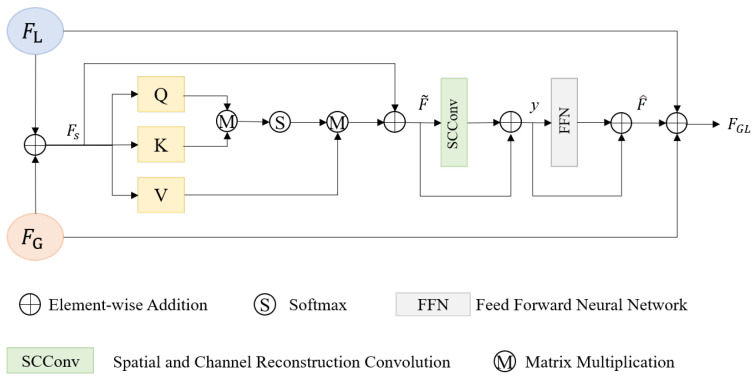
Network structure of proposed MFAF module.

In order to fully utilize local and global features extracted from the dual-encoding network, a multi-feature adaptive fusion module is designed with the self-attention mechanism and the SCConv. Figure 3 illustrates the detailed structure of the proposed MFAF module.

Firstly, the local and global feature maps $F_{L}$ and $F_{G}$ are integrated by element-wise addition operation to obtain preliminary fusion features $F_{s}$, then it is fed into the cross-covariance attention calculating the attention along the feature channels instead of focusing on the feature maps across spatial dimensions. To enhance the representation capacity of the feature map, the residual connection is applied to the preliminary fusion features $F_{s}$ and the features that are enhanced by the cross-covariance attention. The specific operation is as follows:

(1) $$\tilde{F}=Attention\left(Q,K,V\right)+F_{s},$$

(2) $$Attention\left(Q,K,V\right)=V\cdot Softmax\left(Q^{T}\cdot K\right).$$

Further, faced with the redundant features extracted by the former network, the SCConv is carried out to reduce the computation of redundant features and promote the learning of representative features. In addition, the feed-forward neural network is introduced to extract higher-order and more abstract feature representations from raw data through nonlinear transformations of multiple hidden layers. To better capture subtle changes in the data and improve model representation and performance, another two residual connections are integrated into the model after the SCConv and FFN as follows:

(3) $$y=\tilde{F}+SCConv\left(\tilde{F}\right),$$

(4) $$\hat{F}=y+FFN\left(y\right).$$

Finally, the enhanced fusion features after a series of manipulations $\hat{F}$ and the original local and global features are fused by element-wise addition. The specific expression is as follows:

(5) $$F_{GL}=F_{G}+F_{L}+\hat{F}.$$

### 3.4. Decoding Stage

**Figure 4:**
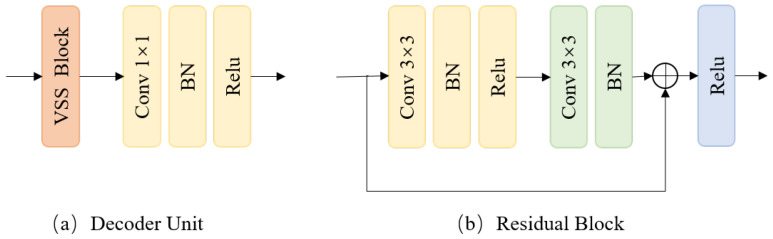
Network structure of decoding stage.

To make full use of the ability to model long sequences and capture long-range dependencies of the Mamba and the capabilities of the CNN model to extract local features, we combine the VSS block and the CNN model to design a decoder unit as shown in Figure 4a. Specifically, the fusion feature maps are fed into a VSS block, followed by a convolution module composed of a 1 × 1 convolution layer, a batch normalization (BN), and a rectified linear unit (Relu) activation function.

In addition, skip connections are introduced to splice features of the same resolution in the corresponding phases of the encoder and decoder in the channel dimension, preserving more detailed information. Further, a residual block is applied to smooth the feature maps and reduce noise, followed by a 1 × 1 convolution to generate the final crack prediction results. The residual block is illustrated in Figure 4b.

### 3.5. Loss Function

The cross-entropy loss function used widely in segmentation is adopted for training, which measures the difference between the probability distribution predicted by the model and the true probability distribution. The specific mathematical equation is illustrated as follows:

(6) $$L=-[ylog\hat{y}+\left(1-y\right)log\left(1-\hat{y}\right)],$$

where *y* denotes the true label and $\hat{y}$ denotes the predicted probability value.

## 4. Experiment Setting

In this section, we provide detailed information on the experimental datasets, which consists of a self-constructed building crack dataset and two widely used public crack datasets, the experimental setting, and the common evaluation metrics.

### 4.1. Datasets

**Figure 5:**
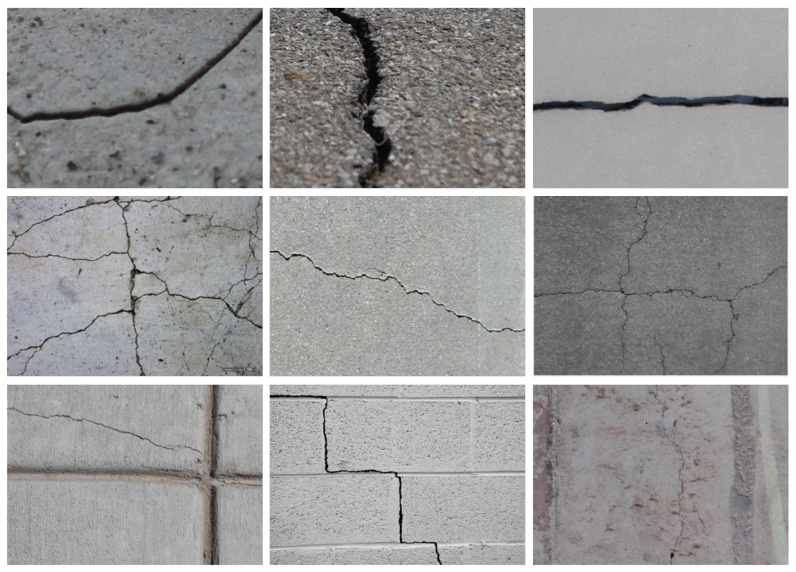
Some examples from the DeepCrack dataset.

**DeepCrack [28]**: This dataset is composed of 537 images of cracks with a resolution of 544 × 384. The cracks in this dataset are diverse and cover a wide range of scenarios including building wall cracks, concrete cracks, and asphalt pavement cracks with various widths and shapes. In the experiment, the training set consists of 477 images and the testing set contains 60 images. Some representative images are shown in Figure 5.

**Figure 6:**
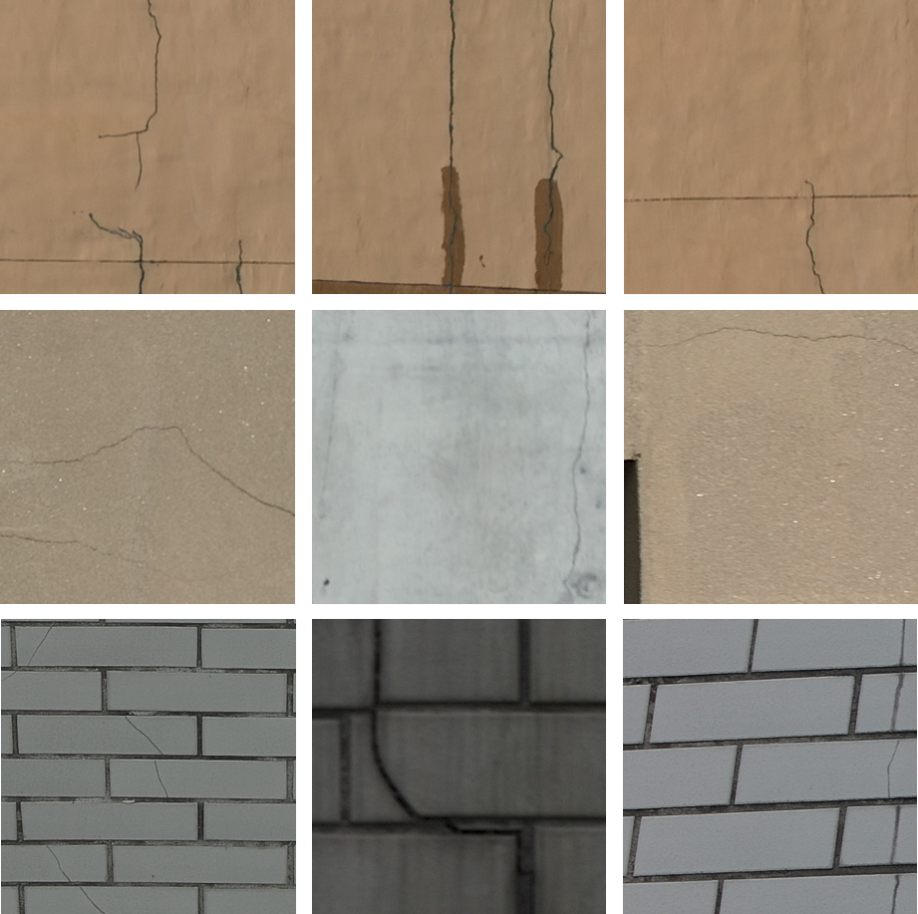
Some examples of the BEWC dataset.

**BEWC dataset**: The dataset of cracks on the exterior walls of buildings captured by drones is sourced from DJI M350RTK drone equipped with the Zenmuse P1 camera, encompassing a wide range of scenarios, such as walls made of tiles, painted walls, and concrete cracks. We select a number of representative buildings and utilize the drone to shoot around them. Specifically, we set the drone 4 m away from the wall and shoot samples every 1.8 m with an image resolution of 5460 × 8192. The crack regions are manually annotated using the semantic segmentation annotation tool Isat-sam which is combined with Meta’s Segment Anything Model (SAM), where the crack areas are labeled in white against a black background. After annotation, the high-resolution drone images are cropped into 512 × 512 pixel images, and pictures containing cracks are selected to form the final dataset of cracks on building exterior walls. Ultimately, 2094 crack images are selected, including 125 painted walls, 662 concrete walls, and 1307 tile walls. Then, it is divided into a training set and a test set for training and testing, respectively. This resulted in a training set of 1671 images and a test set of 423 images. Some representative images are shown in Figure 6.

**Figure 7:**
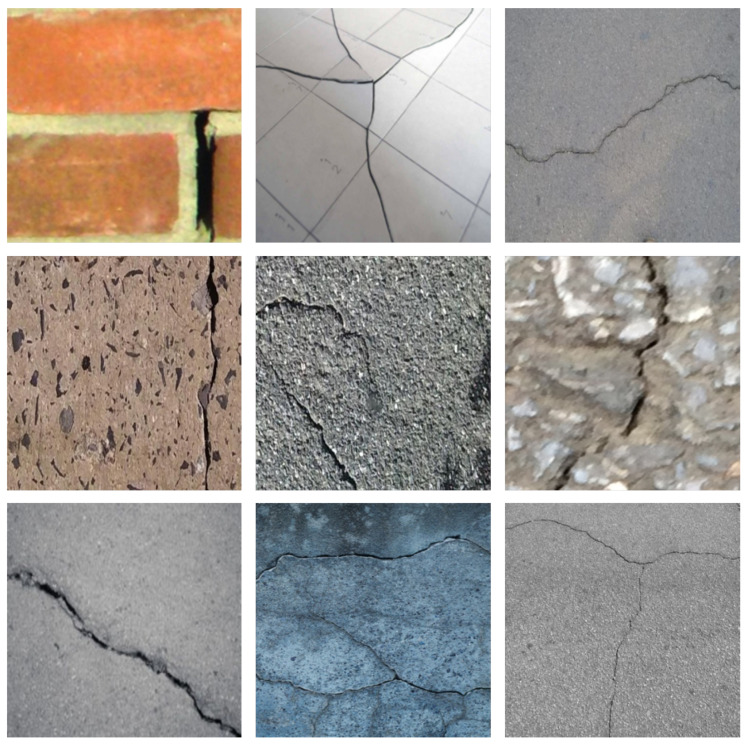
Some examples from the CrackSeg9k dataset.

**CrackSeg9k [43]**: This dataset consists of 9159 images with a resolution of 400 × 400. This dataset is composed of several publicly available datasets, such as crack500 and GAPS384. Due to the inclusion of various types of datasets, it is rich in crack types and has a wide distribution of crack sizes. To increase the training rate, the size of the image is resized to 256 × 256. We divide this dataset according to its default scheme of dividing the training and test sets, and finally obtained 7332 images for the training set and 1827 images for the test set. Some representative images are shown in Figure 7.

### 4.2. Implementation Details

The proposed LGFAF-Net is conducted on the Pytorch framework, and all experiments are implemented on a server equipped with an Nvidia GeForce GTX 3090 GPU. Some experimental hyperparameters are set relying on the researcher’s experience and experimental analysis. During the training stage, the AdamW optimizer with a learning rate of 0.0001 is adopted and the batch size is set to 4. In addition, the model training epoch is set to 500 to allow the model to be fully fitted.

### 4.3. Evaluation Metrics

The widely used evaluation metrics for crack segmentation are Precision, Recall, F1-score (F1), and mIoU. Precision is the ratio of correctly predicted positive samples to all samples predicted as positive, reflecting the false detection rate; Recall is the ratio of correctly predicted positive samples to all true positive samples, reflecting the missed detection rate; F1-score is the harmonic mean of Precision and Recall, which considers both the false detection rate and the missed detection rate and can better reflect the detection ability of the method; IoU is the Intersection over Union between the predicted map and the ground truth map, and mIoU is the average IoU for all categories. The larger these four evaluation metrics are, the better performance will be. The formulas are as follows:

(7) $$Recall=\frac{TP}{TP+FN},$$

(8) $$Precision=\frac{TP}{TP+FP},$$

(9) $$F1=\frac{2\times Precision\times Recall}{Preicision+Recall},$$

(10) $$mIoU=\frac{TP}{FN+TP+FP},$$

where TP is the positive sample with correct prediction. FN is the positive sample with incorrect prediction. FP is the negative sample with incorrect prediction. TN is the negative sample with correct prediction.

## 5. Results and Discussion

In order to fully demonstrate the superiority of the proposed network, the following popular networks used to identify surface damage such as cracks are selected to be compared with our method, including CrackSegNet [30], EMRA-Net [31], Crackformer-II [38], APFNet [32]. Three cracks dataset, consisting of the BEWC dataset produced in this paper and two widely used public cracks datasets DeepCrack and CrackSeg9k, are applied to evaluate the performance of the proposed method. In addition, the ablation study is carried out to evaluate the contribution of each module.

### 5.1. Comparative Experiments

**The results on the DeepCrack dataset**: The objective experimental results of the proposed method on the dataset are presented in Table 1. The proposed network has achieved excellent results in the four objective evaluation indicators previously mentioned, including 89.83% on Precision value, 87.50% on Recall value, 88.65% on F1-score, and 89.40% on mIoU value. Although our method does not achieve the best results on Recall, it outperforms the compared methods on the other three metrics. Specifically, our method exceeds the second-best model EMRA-Net by 1.84% in Precision, 1.21% in Recall, 1.52% in F1-score and 1.26% in mIoU, respectively. In Recall, although our method performs worse than APFNet, our model obtains a gain of 5.15% in Precision, 1.93% in F1-score, and 1.61% in mIoU, respectively, compared with the APFNet.

**Figure 8:**
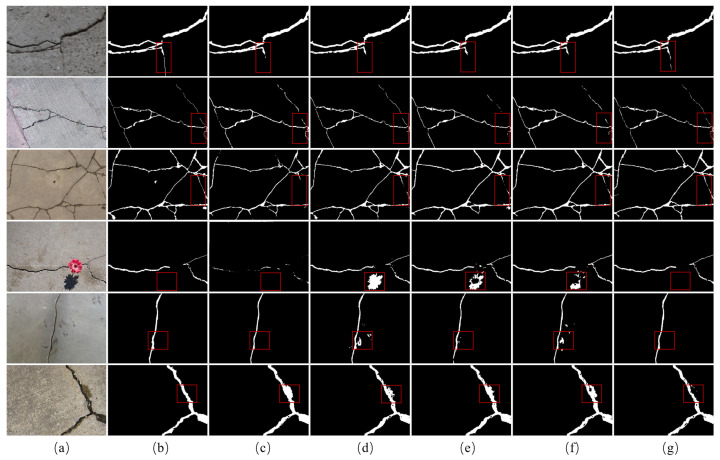
Visualization results of comparison experiments on the DeepCrack dataset. (**a**) Raw image; (**b**) ground truth; (**c**) CrackSegNet; (**d**) EMRA-Net; (**e**) CrackFormer-II; (**f**) APFNet; (**g**) proposed LGFAF-Net. Distinct regions are marked with red boxes.

The visualization results are illustrated in Figure 8. In the first three rows, the areas marked with red boxes are very tiny and indistinguishable from the background. All four comparison networks from Figure 8c–f fail to completely identify the fine cracks while the proposed method exhibits greater proximity to the ground truth. In the fourth to sixth rows, the areas are labeled with red boxes including the regions that are particularly susceptible to being misidentified as cracks, such as shadows and pits. As shown in Figure 8, the proposed LGFAF-Net eliminates interference and correctly detects the cracks. However, most comparison methods falsely identify the areas similar to cracks but not cracks.

**The results on the BEWC dataset**: The objective experimental results of the proposed method on the BEWC dataset are presented in Table 2. The proposed network achieves excellent results in the four objective evaluation indicators previously mentioned, including 76.75% on Precision value, 77.18% on Recall value, 76.96% on F1-score, and 81.20% on mIoU value. Although our method does not achieve the best results in Precision, it outperforms the compared methods in the other three metrics. Specifically, our method outperforms the second-best model APFNet by 2.71% in Precision, 3.12% in Recall, 2.91% in F1-score, and 1.89% in mIoU, respectively. In Precision, although our method performs worse than CrackFormer-II, our model outperforms CrackFormer-II by 9.04% in Recall, 2.99% in F1-score and 1.94% in mIoU, respectively.

**Figure 9:**
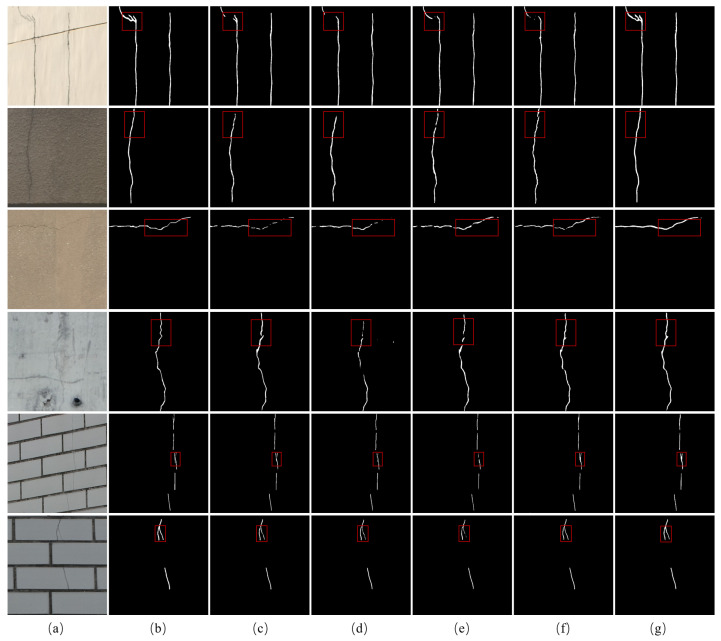
Visualization results of comparison experiments on the BEWC dataset. (**a**) Raw image; (**b**) ground truth; (**c**) CrackSegNet; (**d**) EMRA-Net; (**e**) CrackFormer-II; (**f**) APFNet; (**g**) proposed LGFAF-Net. Distinct regions are marked with red boxes.

The visualization results are shown in Figure 9, which contains various scenes including walls made of tiles, painted walls, and concrete walls. To better compare and observe, we utilize red boxes to mark the location of the differences in segmentation results from different networks. In the first row, it is a long and complex crack in a painted wall. As shown in Figure 9, the proposed LGFAF-Net can effectively identify the complete crack while the comparison networks extract the slender crack but fail to characterize the areas with complex shapes that are labeled with red boxes. For cracks similar to the background which is hard to segment, the proposed method also outperforms other comparison models in terms of the completeness and continuity of crack identification, as shown in Figure 9, the second row to the fourth row. In addition, in the fifth and sixth rows, the proposed network is closer to the ground truth when dealing with the fine cracks in walls made of tiles.

**The results on the CrackSeg9k dataset**: The objective experimental results of the proposed method on the CrackSeg9k dataset are presented in Table 3. The proposed network achieves excellent results in the four objective evaluation indicators previously mentioned, including 76.73% on Precision value, 73.68% on Recall value, 75.17% on F1-score, and 79.36% on mIoU value. Although our method does not achieve the best results in Precision and Recall, it outperforms the compared methods in the other two metrics. Specifically, our method outperforms the second-best model APFNet by 3.08% in Precision, 0.94% in F1-score, and 0.65% in mIoU, respectively. In Precision, although our method performs worse than CrackSegNet, our model outperforms CrackSegNet by 3.2% in Recall, 1.65% in F1-score, and 1.08% in mIoU, respectively.

**Figure 10:**
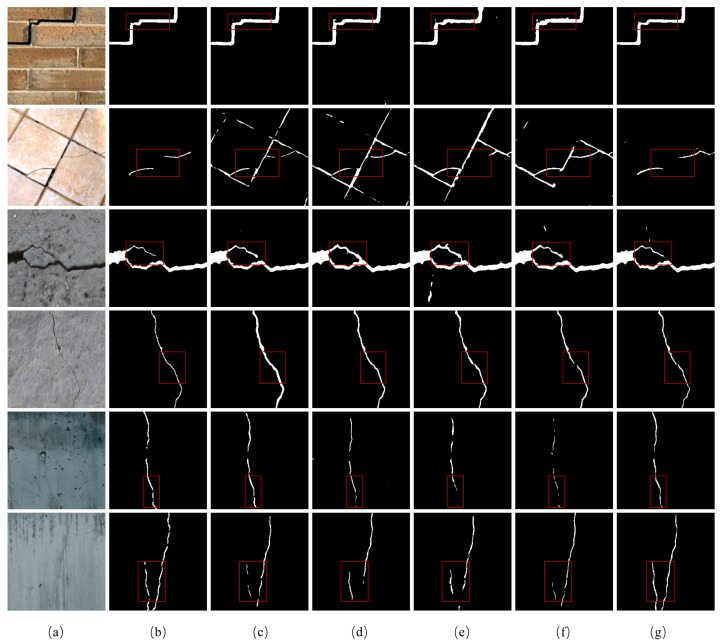
Visualization results of comparison experiments on the CrackSeg9k dataset. (**a**) Raw image; (**b**) ground truth; (**c**) CrackSegNet; (**d**) EMRA-Net; (**e**) CrackFormer-II; (**f**) APFNet; (**g**) proposed LGFAF-Net. Distinct regions are marked with red boxes.

The visualization results are shown in Figure 10, which contains various scenes. To better compare and observe, we utilize red boxes to mark the location of the differences in segmentation results from different networks. In the second row, there is a very obvious interference at the tile joints, which can be easily misidentified as cracks, and the proposed LGFAF-Net accurately identifies the area that is a crack compared to other comparison methods. In the third and fourth rows, for the finer cracks in the red boxes, our method segments them more accurately and closer to the ground truth. In addition, in the last two rows, our method outperforms other comparison models when faced with the case of severe background interference.

The above analysis of the objective evaluation indicators and visualization results confirms that the proposed LGFAF-Net outperforms other comparison methods, which demonstrates the combination of the Mamba and the CNN, and the multi-feature adaptive fusion module are effective in crack detection and can improve the accuracy of crack segmentation.

### 5.2. Ablation Experiments

To investigate the effectiveness of each component of the network in crack segmentation, ablation experiments are performed using the DeepCrack dataset as an example. The baseline stands for the network composed of the VMamba encoder and the decoder designed in this paper. On the basis of the baseline network, we sequentially add the CNN encoder branch and the multi-feature adaptive fusion module that fuses the features of both encoder branches to the network.

As shown in Table 4, the best performance is obtained by the model that includes all components while the baseline with nothing performs the worst. Specifically, the proposed LGFAF-Net achieves the best performance with 89.83% in Precision, 87.50% in Recall, 88.65% in F1-score and 89.40% in mIoU. Due to the removal of the CNN encoder branch and the fusion module, the baseline network achieves the result with 88.50% in Precision, 86.40% in Recall, 87.44% in F1-score, and 88.39% in mIoU. It can be observed very clearly from the table that the network’s performance is improved by adding a CNN encoder branch to the baseline. More specifically, compared to the baseline, F1-score improves by 0.8% and mIoU improves by 0.67%. Furthermore, when the proposed MFAF module is added to the dual-encoding network, the best objective evaluation indicators have been achieved. Compared to the baseline, F1-score improves by 1.21% and mIoU improves by 1.01%. The above analysis demonstrates the effectiveness of the combination of the VMamba encoder and the CNN encoder as well as the advancement of the proposed multi-feature adaptive fusion module.

**Figure 11:**
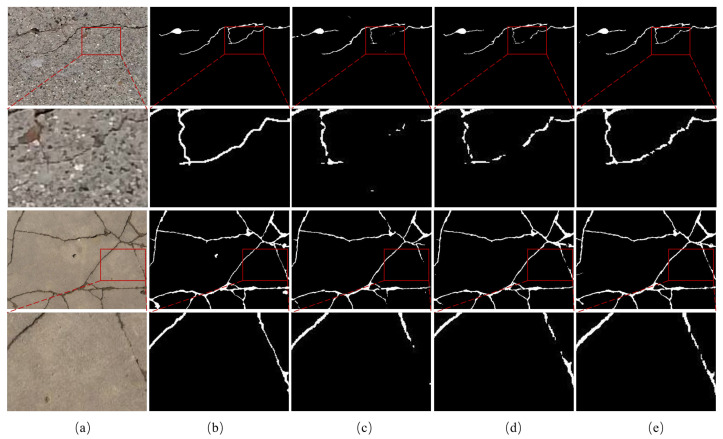
Visualization results of ablation experiments. (**a**) Raw image; (**b**) ground truth; (**c**) baseline; (**d**) baseline + CNN encoder; (**e**) proposed LGFAF-Net.

The visualization results are shown in Figure 11. To observe more carefully, we enlarge the key area. From the third to the fifth column, the segmentation results are approaching the ground truth, which shows that each of our proposed modules is useful for crack segmentation. It can be observed that the baseline has extracted the overall crack area, but has performed poorly for some subtle areas. With the addition of the CNN local feature extraction branch, the detail areas are better recognized. With the addition of the designed MFAF module, the local and global features from the dual branches are better fused to facilitate the learning of the representative feature features, and the segmentation results are closest to the ground truth.

### 5.3. Estimation of Crack Geometry Information

After the cracks have been accurately extracted, the computation of geometric characteristics of the cracks is also essential for the correct assessment of the extent of damage to the building. We use the method based on digital image processing to calculate the length and width of cracks. For the length of cracks defined as the length of the skeleton line at the center of the crack, the center skeleton line of each crack is extracted, and then the respective length of each crack is calculated through each skeleton line. For the calculation of the length of the skeleton line, the length of the whole crack can be calculated by sequentially calculating the distance between two neighboring pixel points and summing them. The distance $p_{k}$ between two neighboring pixel points on the skeleton line is as follows:

(11) $$p_{k}=\sqrt{{\left(x_{k+1}-x_{k}\right)}^{2}+{\left(y_{k+1}-y_{k}\right)}^{2}},$$

where $x_{k}$, $y_{k}$ and $x_{k+1}$, $y_{k+1}$ are the pixel coordinates of the kth and (k+1)th pixel point, respectively. The total length of the cracks $L_{p}$ is as follows:

(12) $$L_{p}=\Sigma p_{k}.$$

**Figure 12:**
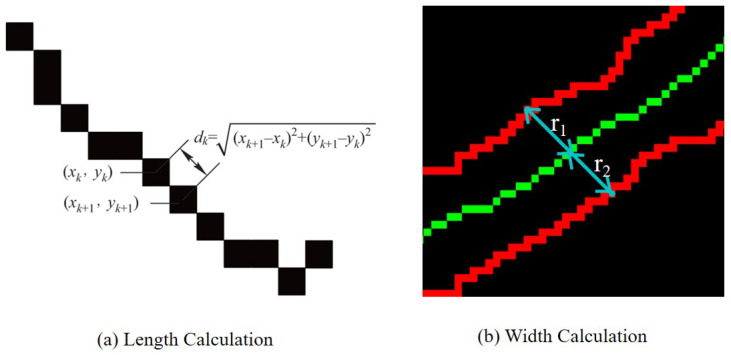
Calculation of crack geometry information. The green line represents the center skeleton line of the crack and the red line represents the edge line of the crack.

For the calculation of crack width, a method based on the central skeleton line is adopted. Firstly, this method requires obtaining the edge lines and central skeleton lines of the cracks. For a point on the central skeleton line of the crack where the width is to be determined, a perpendicular line from this point to the central skeleton line is drawn. The perpendicular line intersects with the two edge lines at respective intersection points. The sum of the distances from the point on the central line where the width is to be determined to the intersection points on the edge lines is the crack width at that point, which is as follows:

(13) $$W_{p}=r_{1}+r_{2},$$

where $r_{1}$ and $r_{2}$, respectively, represent the distance from the center point to the edge lines on both sides. The schematic diagram of length and width calculation is shown in Figure 12.

**Figure 13:**
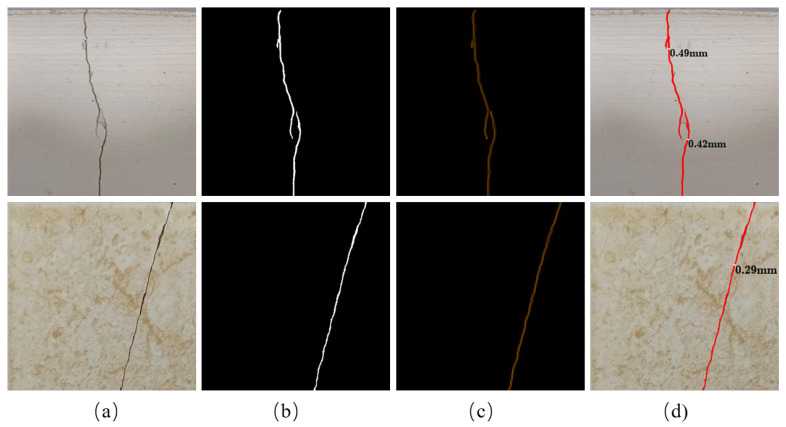
Some examples of estimation of crack geometry information. (**a**) Row image; (**b**) ground truth; (**c**) the edge lines and central skeleton lines; (**d**) crack width.

We chose to take some test images indoors to verify the usability of our method. We placed a ruler in the shot as a scale so that we could deduce the actual distance corresponding to one pixel in the image based on the actual length of the ruler *L* and the number of pixels it occupied *P*, and the spatial resolution of the image which is usually expressed in terms of ground sampling distance (GSD) is calculated as follows:

(14) $$GSD=\frac{L}{P},$$

where GSD is the ground sampling distance, which is the actual distance corresponding to one pixel. The two sets of crack images and the results of crack identification are shown in Figure 13. The pixel length and width of the cracks, as well as the actual length and width converted based on the corresponding GSD, are presented in Table 5 and the specific calculation process is as follows:

(15) $$L_{a}=L_{p}\times GSD,$$

(16) $$W_{a}=W_{p}\times GSD,$$

(17) $$W_{e}=\left|W_{r}-W_{a}\right|,$$

where $L_{p}$ represents the pixel length of the crack, $L_{a}$ s the calculated actual length of the crack, $W_{p}$ represents the pixel width of the crack, $W_{a}$ is the calculated actual width of the crack, $W_{r}$ is the real width of the crack measured with a crack meter, $W_{e}$ is the error of crack width identification, and GSD is the ground sampling distance, which is the actual distance corresponding to one pixel. For these two sets of images, GSD is 0.098 mm/pixel, which means the actual distance corresponding to one pixel is 0.098 mm. The visualization process is shown in Figure 13.

According to the above process and formulas, the specific geometric results of three cracks in the two sets of crack images are shown in Table 5. It can be seen that the cracks are completely identified, and the actual length and width of the cracks are also calculated accordingly. For the crack width, the error of this method is controlled within 0.1 mm, which can obtain relatively accurate actual widths of the cracks.

### 5.4. Impact of Crack Characteristics on Structural Health

Crack characterization is a complex but vital matter for the analysis and evaluation of structural health, which involves several aspects of spatial size, depth, shapes, and others.

**The spatial size of cracks**: This includes the length and width of cracks. Shorter cracks may only affect localized areas, while long cracks may run through the entire structure, seriously affecting its load-bearing capacity and stability. Generally speaking, cracks with widths less than 0.2 mm may not affect the structural safety of the building, and cracks with widths above 0.2 mm need regular inspections and the changes in the cracks should be recorded [44,45]. Among them, for cracks with larger widths, further risk assessment is required to determine whether repair measures are needed.

**The depth of cracks**: Methods for crack depth detection include thermal imaging techniques [46], stress wave theory [47], and ultrasonic techniques [48]. As the crack depth increases, the structure becomes more prone to deformation and damage when subjected to external forces, thereby affecting its load-bearing capacity. The deeper the crack is, the more likely it is to cause exterior wall leakage and accelerate steel corrosion, thereby endangering structural health.

**The shape of cracks**: The shape of cracks can be categorized into linear, curved, and alligator [49,50]. Straight-line cracks usually extend along a certain direction, including longitudinal and transverse cracks, which have a certain effect on the structural integrity; arc-shaped cracks indicate that there is a large local stress concentration in the structure; and alligator cracks may reduce the structural integrity and durability.

**Others**: Other factors affecting structural safety include the distribution and the development trend of cracks. We should pay attention to whether the cracks appear in the critical stress areas of the structure, such as the nodes of beams and columns, or the weak areas of the walls. In addition, the cracks should be observed to assess whether they are expanding, or deforming, which may pose a greater risk to structural safety.

In summary, various characteristics of cracks are crucial to structural health. In this paper, we mainly focus on one important factor about the buildings of spatial size, where we attempt to propose a crack detection algorithm based on UAV data. By integrating the results of image-based crack detection with UAV image positioning information, this algorithm can assist in initially screening building areas that may pose potential safety hazards, thereby reducing manual workload and improving detection efficiency. In the future, determining how to further obtain the depth of cracks through the multimode sensors (LIDAR, infrared, etc.) carried by UAV needs to be further explored.

## 6. Conclusions

The proposed LGFAF-Net aims to detect cracks in building walls accurately. The proposed network combines the VMamba encoder and the CNN encoder to utilize the ability of the Mamba to model global dependencies and the CNN network to capture local features. In addition, to better fuse the features extracted from the dual-encoding network, the MFAF module is introduced, which can facilitate representative feature learning and improve the accuracy of the crack segmentation. Furthermore, a building exterior wall crack dataset captured by UAVs is built in this paper, making up for the lack of data on building cracks. Finally, we have conducted extensive experiments on both self-constructed building crack datasets and widely used crack public datasets to demonstrate the effectiveness and advancement of our proposed methodology. In our future work, we will utilize the information from thermal infrared images to further detect the crack depth features and realize the lightweight to be deployed on edge devices for real-time detection, in order to achieve a more comprehensive exploration of the crack features to ensure the safety of the building structure.

## Figures and Tables

**Table 1:** Comparative results for different methods on the DeepCrack dataset.

	Precision (%)	Recall (%)	F1-Score (%)	mIoU (%)
CrackSegNet [30]	85.73	87.05	86.38	87.52
EMRA-Net [31]	87.99	86.29	87.13	88.14
CrackFormer-II [38]	84.90	87.95	86.40	87.53
APFNet [32]	84.68	**88.86**	86.72	87.79
LGFAF-Net	**89.83**	87.50	**88.65**	**89.40**

**Table 2:** Comparative results for different methods on the BEWC dataset.

	Precision (%)	Recall (%)	F1-Score (%)	mIoU (%)
CrackSegNet [30]	79.50	68.39	73.53	78.98
EMRA-Net [31]	75.40	72.58	73.97	79.25
CrackFormer-II [38]	**80.89**	68.14	73.97	79.26
APFNet [32]	74.04	74.06	74.05	79.31
LGFAF-Net	76.75	**77.18**	**76.96**	**81.20**

**Table 3:** Comparative results for different methods on the CrackSeg9k dataset.

	Precision (%)	Recall (%)	F1-Score (%)	mIoU (%)
CrackSegNet [30]	**76.83**	70.48	73.52	78.28
EMRA-Net [31]	73.88	71.13	72.48	77.59
CrackFormer-II [38]	72.49	73.29	72.89	77.83
APFNet [32]	73.65	**74.82**	74.23	78.71
LGFAF-Net	76.73	73.68	**75.17**	**79.36**

**Table 4:** Ablation experiments for the proposed methods on the DeepCrack dataset.

	Precision (%)	Recall (%)	F1-Score (%)	mIoU (%)
Baseline	88.50	86.40	87.44	88.39
Baseline+CNN Encoder	89.69	86.84	88.24	89.06
LGFAF-Net	**89.83**	**87.50**	**88.65**	**89.40**

**Table 5:** Some examples of estimation of crack geometry information.

		$L_{p}$ (pixel)	$L_{a}$ (mm)	$W_{p}$ (pixel)	$W_{a}$ (mm)	$W_{r}$ (mm)	$W_{e}$ (mm)
image1	crack1	397.53	38.96	5	0.49	0.45	0.04
	crack2	241.67	23.68	4.29	0.42	0.35	0.07
image2	crack1	515.19	50.49	2.96	0.29	0.25	0.04

## Data Availability

The data are not publicly available due to privacy.
